# Patterns of human herpesvirus-8 oral shedding among diverse cohorts of human herpesvirus-8 seropositive persons

**DOI:** 10.1186/s13027-016-0052-2

**Published:** 2016-02-10

**Authors:** Rachel A. Bender Ignacio, Jason D. Goldman, Amalia S. Magaret, Stacy Selke, Meei-Li Huang, Soren Gantt, Christine Johnston, Warren T. Phipps, Joshua T. Schiffer, Richard A. Zuckerman, R. Scott McClelland, Connie Celum, Larry Corey, Anna Wald, Corey Casper

**Affiliations:** Vaccine and Infectious Diseases Division, Fred Hutchinson Cancer Research Center, Seattle, WA USA; Division of Allergy and Infectious Diseases, Department of Medicine, University of Washington, Seattle, WA USA; Department of Epidemiology, School of Public Health, Seattle, WA USA; Department of Biostatistics, School of Public Health, Seattle, WA USA; Department of Laboratory Medicine, University of Washington, Seattle, WA USA; Division of Infectious Diseases, Department of Pediatrics, University of British Columbia, Vancouver, BC Canada; Section of Infectious Disease and International Health, Department of Medicine, Geisel School of Medicine at Dartmouth, Lebanon, NH USA; Department of Global Health, University of Washington, Seattle, WA USA

**Keywords:** Herpesviridae infections, Herpesvirus 8, Human, Kaposi sarcoma-associated herpesvirus (KSHV), Sarcoma, Kaposi, HIV infections, Virus replication

## Abstract

**Background:**

Human herpesvirus-8 (HHV-8), the etiologic agent of Kaposi sarcoma (KS), establishes lifelong latent infection with periodic lytic replication (“shedding”) at mucosal sites, especially the oropharynx. Patterns of HHV-8 shedding are not well understood, and require elucidation to better predict risk of HHV-8 related malignancies in those infected. We sought to characterize patterns of HHV-8 oropharyngeal shedding among diverse cohorts that enrolled HHV-8 seropositive persons.

**Methods:**

We quantified HHV-8 oral shedding using PCR among HHV-8 seropositive persons who collected at least 14 days of oral swabs in 22 studies on 3 continents. We excluded persons taking antivirals during sampling or any prior use of antiretrovirals in those who were HIV-infected.

**Results:**

248 participants were enrolled from the US, Peru, Cameroon, Uganda, and Kenya; 61 % were men, 58 % were HIV seropositive, and 16 % had KS. Overall, 3,123 of 10,557 samples (29.6 %) had HHV-8 detected. Quantity of virus shed was highly correlated with shedding rate, (ρ = 0.72, *p* < 0.0001). HHV-8 was detected in ≥1 sample in 55 % of participants with a median of 7 % of days in the US and Kenya, 0 % in Uganda and Peru, and 18 % in Cameroon. Median episode duration was three days, and episodes with high median quantity lasted longer (42 vs 3 days, *p* < 0.0001). In persons with multiple observations over time, 66 % of shedding rate variance was attributable to differences between individuals.

**Conclusions:**

In HHV-8 infected individuals from diverse settings, oral mucosal shedding rate, quantity, and duration were correlated; individual shedding was highly variable. Studies are needed to determine factors accounting for between-person variation and the relationship of HHV-8 shedding to development of associated diseases.

## Background

Human herpesvirus-8 (HHV-8), also known as Kaposi sarcoma-associated herpesvirus (KSHV), establishes lifelong latent infection, punctuated with periods of lytic replication at mucosal sites (“shedding”) [1]. Shedding is most commonly detected at the oropharynx, which is thought to be the site of transmission, primary HHV-8 latency and reactivation [2–4]. Prevalence of HHV-8 infection is geographically variable, in >60 % of adults in Uganda and other sub-Saharan African (SSA) countries [5, 6] and as low as 3.3 % in healthy Americans [7, 8]. While in most people HHV-8 is asymptomatic, HHV-8 replication is considered to be integral to the development and maintenance of Kaposi sarcoma (KS). Other sequelae of the infection include Multicentric Castleman Disease (MCD), Primary Effusion Lymphoma as well as the recently described KHSV Inflammatory Cytokine Syndrome [9–12].

Despite high seroprevalence of HHV-8 in many regions, HHV-8 associated diseases are relatively uncommon, except in the endemic regions of SSA, or in persons with advanced immunosuppression. KS remains the second most prevalent cancer in men and the fifth most prevalent cancer in women in the WHO Africa region [13]. Previous work by our group demonstrated that HHV-8 mucosal shedding is associated with presence of KS and male sex, but not with HIV infection [4]. Prior studies assessing oral HHV-8 shedding evaluated small numbers of participants and were limited in geography [14]. The patterns of HHV-8 shedding, such as duration of shedding episodes, interval between episodes, and variability between persons have not yet been characterized. Due to the heterogeneity of cohort composition, we describe shedding characteristics and patterns across all persons, avoiding prediction of shedding by geographic, demographic or clinical covariates. We assembled diverse cohorts of HHV-8 infected individuals to describe patterns of HHV-8 oral mucosal shedding including rate, quantity, duration, and within- versus between-person variability over time.

## Results

### Study participants

**Fig. 1:**
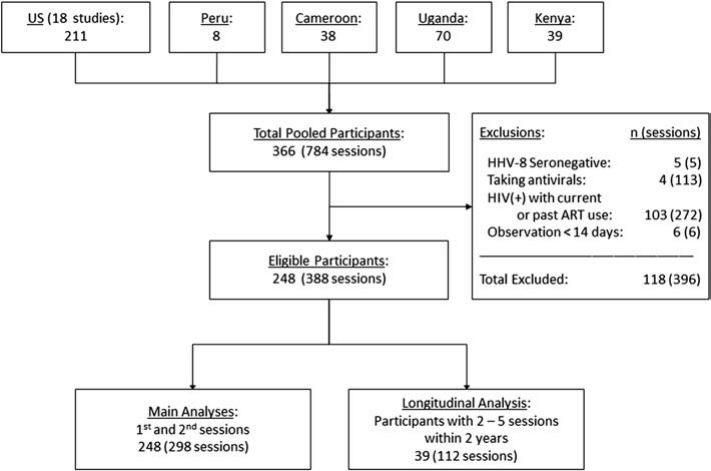
Flow diagram of study participant inclusion. Numbers listed are n = number of participants (“sessions” = number of observation periods contributed). Abbreviations: ART, antiretroviral therapy; US: United States

**Fig. 2:**
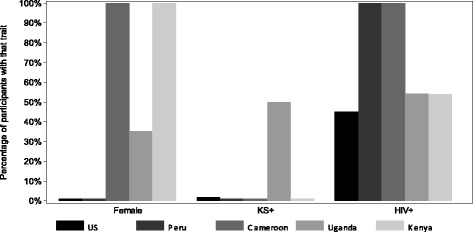
Heterogeneity of participant characteristics within the cohorts included in this study

Between 1993 and 2011, 248 subjects met inclusion criteria and contributed 298 sessions (observation periods) to the primary analysis, with median duration of 30 days (range 14–136) per session [Fig. 1]. These cohorts included persons at high risk for HHV-8 infection, including heterosexuals from KS-endemic regions, men who have sex with men, and female sex workers. Overall, 152 (61 %) participants were men, and the median age was 38 (interquartile range, IQR: 31–45). One hundred forty-three participants (58 %) were HIV-infected, 35 (14 %) had KS, and 1 (0.4 %) had MCD. Among 143 persons with HIV, CD4 count was <200 in 9 (6 %), 200–499 in 31 (22 %), ≥500 in 17 (12 %) and not available in 86 (60 %). HIV RNA was <10^4^ copies/mL in 23 (16 %), and unmeasured in 70 (49 %). Sixteen (44 %) had endemic KS, and 20 (56 %) had HIV-associated KS. All participants from Peru and Cameroon were HIV-infected, while other sites enrolled both HIV-infected and uninfected persons [Table 1 and Fig. 2].

**Table 1:** Cohort, participant, and shedding characteristics

Participant Location, N (%)^a^	USA	Peru	Cameroon	Uganda	Kenya	Total
	102 (41 %)	7 (3 %)	32 (13 %)	68 (27 %)	39 (16 %)	248 (100 %)
Participant Demographic and Clinical Data:						
Male, n (%)	101 (99 %)	7 (100 %)	0 (0 %)	44 (65 %)	0 (0 %)	152 (61 %)
HIV-infected, n (%)	46 (46 %)	7 (100 %)	32 (100 %)	37 (54 %)	21 (54 %)	143 (58 %)
CD4 count^b^						
CD4 < 200	9 (20 %)	0 (0 %)	0 (0 %)	--	--	9 (6 %)
CD4 200–499	17 (37 %)	1 (14 %)	13 (41 %)	--	--	31 (22 %)
CD4 500+	12 (26 %)	0 (0 %)	5 (16 %)	--	--	17 (12 %)
Unknown	8 (17 %)	6 (86 %)	14 (44 %)	37 (100 %)	21 (100 %)	86 (60 %)
HIV-1 RNA^b^						
<10^4^	12 (26 %)	4 (57 %)	--	7 (18 %)	--	23 (16 %)
≥10^4^	22 (48 %)	3 (43 %)	--	25 (68 %)	--	50 (35 %)
Unknown	12 (26 %)	0 (0 %)	32 (100 %)	5 (14 %)	21 (100 %)	70 (49 %)
Kaposi sarcoma:	2 (2 %)	0 (0 %)	0 (0 %)	34 (50 %)	0 (0 %)	36 (15 %)
HIV-associated:	1 (1 %)	--	--	19 (28 %)	--	20 (8 %)
HSV-2 seropositive, n/tested (%)	62/97 (64 %)	7 (100 %)	31 (97 %)	ND	ND	100/135 (74 %)
Cohort Shedding Characteristics:						
Sessions,						
Number:	152	7	32	68	39	298
Observations per session:						
Median # of days (range)	33 (15–136)	56 (27–57)	19 (14–23)	29 (14–32)	29 (26–29)	30 (14–136)
Overall shedding:						
Positive swabs:	2,213	75	183	389	263	3,123
Total swabs:	6,660	359	590	1834	1,114	10,557
Percent positive:	(33 %)	(21 %)	(31 %)	(21 %)	(24 %)	(30 %)
Participant shedding rate per session,						
Median % (IQR)	7 (0, 73)	0 (0, 33)	18 (0, 56)	0 (0, 34)	7 (0, 43)	4 (0, 54)
Persons with any shedding	60 (59 %)	3 (43 %)	21 (66 %)	26 (38 %)	27 (69 %)	137 (55 %)
Quantity, median (IQR) of positive swabs, log_10_ copies/mL	4.8 (3.9–5.6)	7.4 (5.0–7.8)	3.2 (2.7–3.8)	5.2 (3.9–6.0)	4.4 (3.5–5.5)	4.7 (3.7–5.6)

*Abbreviations. US* United States, *ND* Not determined, *IQR* Interquartile Range, *HSV* Herpes simplex virus

^a^Percentages are out of those within the given country

^b^Percentages for CD4 count and HIV RNA are of HIV-infected participants

### HHV-8 shedding rate and quantity

Overall, 10,557 swabs were analyzed for HHV-8 DNA, and HHV-8 was detected in 3,123 (29.5 %). Within the entire cohort, 137 persons (55 %) had HHV-8 DNA detected at least once. The median (IQR) shedding rate per session was 7 % (0–73 %) in the US, 0 % (0–33 %) in Peru, 18 % (0–56 %) in Cameroon, 0 % (0–34 %) in Uganda, 7 % (0–43 %) in Kenya, and 4 % (0–54 %) overall [Table 1]. Among US participants, 33 % of swabs had detectable HHV-8 DNA, 21 % in Peru, 31 % in Cameroon, 21 % in Uganda and 24 % in Kenya [Table 1]. In order to validate the utilization of samples from persons with and without HIV and KS, we compared shedding rate within the factorial design of the Ugandan cohort as previously described [4]. In 34 Ugandan persons with KS, the shedding rate was 25 % in HIV-associated cases and 40 % in endemic KS cases; Ugandans without KS had a shedding rate of 27 %. The Ugandan cohort was the only African cohort to include both men and women; HHV-8 was detected in 30 % of samples from men, compared with 6 % from women. Amongst all cohorts, HHV-8 was detected in 18 % of swabs from HIV-infected individuals and 18 % of swabs from HIV-uninfected individuals.

**Fig. 3:**
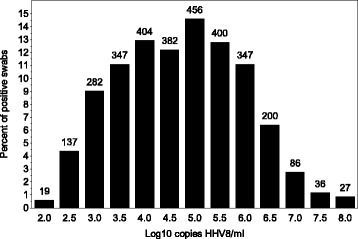
Quantity of HHV-8 DNA (Log_10_ copies/mL) within swabs positive for HHV-8. 2.17 Log_10_ copies/mL represents the lower limit of accurate detection (150 copies/mL). The label for each bin represents a range including the value listed and all values up to but not including the value listed in the next highest bin

**Fig. 4:**
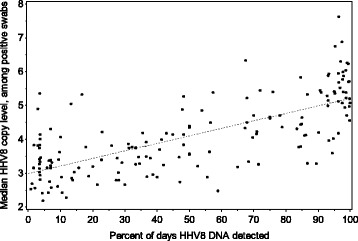
Correlation of HHV-8 DNA quantity and shedding rate: Scatterplot of HHV-8 quantity (median HHV-8 DNA copy number by qPCR during shedding session) vs. HHV-8 shedding rate (number of days HHV-8 is detectable at any level by qPCR divided by number of days of shedding session). Best-fit regression line is shown (ρ = 0.72, *p* < 0.0001)

The median quantity of HHV-8 DNA in samples with HHV-8 detected was 4.7 (IQR: 3.7–5.6) log_10_ copies/mL [Fig. 3]. Median HHV-8 quantity during the session correlated strongly with shedding rate (ρ = 0.72, *p* < 0.0001) [Fig. 4]. Maximum quantity of HHV-8 detected during a session also correlated highly with the shedding rate (ρ = 0.82, *p* < 0.0001) (data not shown).

### Shedding episodes

We defined a shedding episode as a period of detectable HHV-8 of at least one day bounded by at least two preceding and subsequent negative swabs, and could include singular missed or negative swabs [15]. When initiation or termination of the shedding episode was not observed, interval-censoring survival was used estimate the episode duration. We dichotomized episodes by median quantity above or below the median quantity of all detectable samples and tested the association between quantity and duration for episodes with log rank tests. We evaluated up to 3 shedding episodes per session, for a total of 229 episodes in 116 persons. Duration was uncertain in 106 (46 %) episodes because of censoring at either limit of session observation. For 123 episodes of certain duration, the median duration was 1 day (IQR: 1–4 days). Using interval censoring survival analysis to include both episodes of certain and uncertain duration, 30 % of episodes were predicted to last less than 1 day, 50 % to last less than 9 days, and 60 % to last less than 20 days. One hundred-nineteen episodes (52 %) with a high median quantity (>4.5 log_10_ copies/mL) had a predicted median duration of 42 days; for episodes with lower median quantity (≤4.5 log_10_ copies/mL), the median duration was 3 days (*p* < 0.0001). We investigated the duration of shedding episodes and intervals without HHV-8 shedding between episodes. “Short” episodes were defined as ≤ 3 days (median duration of all episodes) and “long” episodes as > 3 days. Short episodes were preceded by a median 13-day interval without shedding whereas long episodes were preceded by a median 7-day interval (*p* = 0.035). A sensitivity analysis reclassifying episodes censored prior to 3 days (26 episodes) as “short” instead of “long” provided similar estimates.

**Fig. 5:**
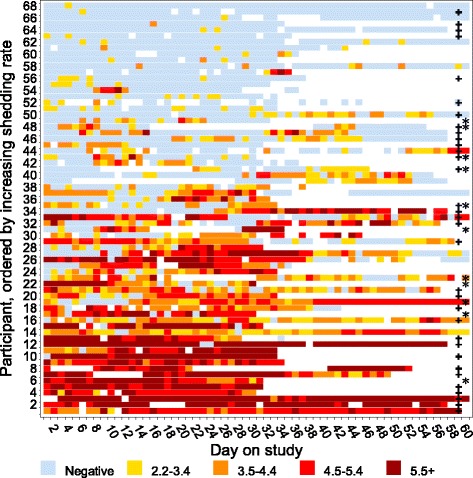
Patterns of HHV-8 Shedding. Included participants had HHV-8 detectable by PCR on at least one day during the first 60 days of observation and were observed for a minimum of 30 days. Each row represents one participant, ordered by shedding rate from lowest to highest over the shedding session. Sixty participants with at least 30 days of observation who never shed were omitted. Time is represented on the x-axis (in days) such that each box represents the HHV-8 PCR result from a single daily swab for an individual. DNA quantity is graded by color (in log_10_ copies/mL). Asterisks (*) denote participants with Kaposi Sarcoma and plus signs (+) denote participants with HIV. White indicates no swab collected; other colors as per legend

To illustrate patterns of HHV-8 shedding, data from participants who collected swabs for 30–60 days and who shed at least once were included in a heatmap [Fig. 5]. We did not observe a singular pattern of shedding, but rather a gradation was noted from infrequent and low copy number shedding to very frequent and high copy number shedding. For example, some participants shed virus in single-day episodes, while others had intermittent episodes of both high and low quantities, and a few participants shed virus at high quantities throughout the session. Participants with KS and HIV were dispersed amongst participants when sorted by shedding rate [Fig. 5].

### Variation in shedding rate over time

**Fig. 6:**
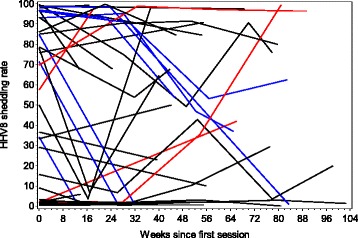
Variation in oral shedding rate of HHV-8 by participant across multiple shedding sessions. All cohort participants with 2–5 sessions within two years were included in the analysis of shedding variation over time, representing 39 participants with 112 total sessions. Four persons had shedding rates increase by at least 30 % on an absolute scale over time (red), 7 persons had rates decrease by the same amount (blue), and 28 had no consistent pattern (black)

Stability of shedding rate over time was evaluated in participants contributing multiple sessions. Thirty-nine participants, all of whom were from the United States, had at least one additional session within two years of the first: 19 persons had 2, 9 persons had 3, 8 persons had 4, and 3 persons had 5, totaling 112 sessions. The median shedding rate of first sessions was 50 %. Over time, 4 persons (10.3 %) had shedding rate increase by at least 30 % on an absolute scale, 7 persons (18.0 %) had shedding rate decrease by the same amount, and 28 (71.8 %) had no consistent pattern or a stable shedding rate [Fig. 6]. Using variance components, we examined the variation in shedding rates over time within the population that was due to inter-person versus intra-person differences. We found that 66 % of the variance was due to inter-person differences and the remainder due to change in an individual’s shedding rate over multiple sessions.

## Discussion

This is the largest study of HHV-8 shedding published to date, evaluating more than 10,000 oral swab samples collected from geographically and demographically diverse HHV-8 seropositive persons not receiving antiviral or antiretroviral therapy. HHV-8 shedding was a common event, although nearly half of participants never shed while under observation. We found a strong correlation between shedding rate, quantity, and duration of oropharyngeal shedding within an individual, but significant variability between individuals.

Our findings are consistent with earlier studies of HHV-8 oropharyngeal shedding that observed 15 %-32 % shedding rates [2, 16, 17]. In our large cohort, we observed a gradation in shedding rate and quantity across participants. In comparison to HSV-2, a herpesvirus with well-characterized shedding patterns, we found several differences in mucosal reactivation patterns. HSV-2 reactivation is characterized by shorter episodes with 20 % lasting >9 days [18], whereas we found longer episodes of HHV-8 shedding, with 40 % ongoing at 20 days. In contrast to HSV-2, which has rapid expansion and decay, we observed variable patterns of HHV-8 shedding, with many episodes showing a long plateau without observed termination. Additionally, quantity of HHV-8 detected tended to be more homogeneous compared to HSV-2, with the vast majority of swabs clustered around 4.5 log copies/mL, whereas HSV-2 is frequently detected at both lower and much higher quantities [18, 19]. Our analysis found similar shedding rates in HIV-infected and uninfected individuals, an intriguing finding that is dissimilar to other herpesviruses, including HSV, CMV, and EBV [20–24].

HHV-8 shedding in relationship to KS and HIV has been studied and results are inconsistent. In Uganda, KS patients were more likely to have HHV-8 detected than those with asymptomatic HHV-8 infection, while HIV serostatus was not associated with detection of HHV-8 in the oral mucosa [4]. Another cross-sectional study found no difference in detection of HHV-8 shedding in the oral mucosa of HIV-infected men with and without KS [25]; persons with KS in our study were not observed prior to onset of disease. Our data do not address the risk of asymptomatically-infected persons developing HHV-8-associated diseases, nor compare shedding patterns between persons with HHV-8 related diseases and asymptomatic infection; the understanding of viral shedding patterns gained from our work may be used to design longitudinal studies to address questions of HHV-8 transmission and disease association.

Our study was limited by performance characteristics of the HHV-8 serologic assays used to include and exclude participants, which have sub-optimal sensitivity and specificity [26, 27]. We may have excluded persons from our cohort who were HHV-8 infected but seronegative, or included those with false positive serology. Using PCR to quantitate oral virus may not strictly detect lytic replicating virus, as latent cell-associated virus may also be present in these samples. However, that the large majority of samples were negative in HHV-8 seropositive persons suggests that latent virus in oral epithelial cells is generally not detected by this collection method and PCR technique. Additionally, we were limited in our ability to model demographic and clinical risk factors for HHV-8 shedding due to both measured and unmeasured cofounders in these heterogeneous cohorts, especially with respect to available HIV clinical data. Conversely, the diversity of participants in this large cohort allowed us to describe HHV-8 shedding patterns unrestricted to any narrow population or geographic area, as has frequently been done up to this point. We benefitted from long sessions and serial observations on many participants; prior studies have not been able to evaluate viral replication patterns in individuals over time. Other strengths of this study include robust methods of swab collection, laboratory, and statistical analysis concordant with methods used by our group in studies of herpesvirus shedding over the last three decades. While different PCR methods were used in our reference laboratory during the time period over which our cohorts span, the standardization was consistent across all cohorts, was supervised by the same virologist, and quality control showed high correlation between assays.

## Conclusions

We did not detect a consistent or singular pattern to HHV-8 shedding, and substantial variability was observed between persons. We demonstrated that rate and quantity of HHV-8 shedding are highly correlated and that persons experiencing longer and higher quantity shedding episodes can be predicted to shed more frequently in the future. Patterns of HHV-8 shedding are distinctly different from other well-characterized herpesvirus infections, such as HSV-2. This work provides a foundation to assess mucosal shedding of HHV-8 as a possible biomarker to assess risk of HHV-8 transmission or disease. Longitudinal studies in asymptomatically infected persons are needed to further understand clinical correlates to patterns of HHV-8 shedding, and if replication is associated with HHV-8 related diseases.

## Methods

We evaluated 22 prospective cohorts in which HHV-8 DNA quantification had been performed on oropharyngeal swabs, including 18 cohorts from the Virology Research Clinic, University of Washington [14, 28–32], and 4 studies by affiliated investigators in Lima, Peru [33]; Kampala, Uganda [4]; Mombasa, Kenya [34]; and Yaounde, Cameroon. The University of Washington Institutional Review Board and appropriate partner institutions for international sites approved each study; all participants provided written informed consent in accordance with the Declaration of Helsinki. Participants collected daily samples of the oropharyngeal mucosa with a Dacron swab (three times weekly in Cameroon only) with similar technique across all studies, for a minimum of 14 days [35]. Each such observation period is termed a “session”. We included HHV-8 seropositive adults (age ≥18) with and without HIV, and with and without HHV-8 associated diseases including MCD and KS. We included HIV-infected participants only if antiretroviral naïve to avoid confounding from immune restoration and potential direct and indirect effects of ART on HHV-8 replication [36]. We excluded participants receiving antiviral therapies including valganciclovir, acyclovir, valacyclovir or famciclovir [31], and participants with ≤14 samples per session, as prior analyses performed by our group demonstrate substantial instability in herpesvirus shedding estimates with fewer samples [37]. Data from 216 of 298 HHV-8 shedding sessions (72 %) included in this analysis were previously published [1, 4, 14, 16, 31, 34, 38]. We aggregated persons from these diverse populations to more robustly describe associations between shedding rate, quantity, duration, and variability of shedding episodes in HHV-8 infected persons.

### Laboratory methods

HHV-8 seropositivity was determined by positive test result on either immunofluorescence assay (IFA) against latent or lytic antibodies [39] or whole-virus enzyme immunoassay (EIA) [27]. Swabs were collected into lysis buffer containing 100 mM KCl (or NaCl), 25 mM EDTA (pH 8.0), 10 mM Tris (pH 8.0), and 1 % Igepal CA-630 and stored at −20 °C until testing, a process validated to be stable for extended storage [40, 41]. Quantitative polymerase chain reaction (qPCR) was performed to quantitate HHV-8 DNA using previously described and validated methods [1, 16, 38, 42]. Three qPCR assays were used using the study period: the KS330Bam_233_ region of *orf26* (a viral capsid protein)^1^, *orf73* (latency-associated nuclear antigen-1)^2^ or a multiplex assay using *orf73* plus *T07-K12* (kaposin)^3^. Briefly, early samples (orf26 assay) were extracted from swabs with traditional phenol-chloroform method and all other samples were extracted using Qiagen column (QIAmap DNA blood kit), then purified, and amplified with the given primers-probe sets on the Taqman qPCR platform (Applied Biosystems, Foster City, CA). Compared to qPCR with *orf73* as the gold standard, *orf26* had sensitivity 83.1 %, specificity 98.8 %. To minimize the variance in sensitivity at low end of detectable range, a cut-off of ≥150 copies/mL (≥3 copies/PCR reaction) were considered positive for HHV-8 detection [43]. Applying this cut-off, the correlation between orf26 and orf73 results was R^2^ = 0.75. Compared to qPCR with *orf73* and *T07-K12* multiplex as the gold standard, *orf73* had a sensitivity of 94.1 %, specificity of 97.5 %. Applying the minimum cut-off for positivity, the correlation between the latter two primer sets was R^2^ = 0.96. Quantitation standard using the cloned PCR product of KS330Bam_233_ primers was consistent throughout all cohorts. Quality control using standardization and positive and negative controls for each batch were supervised by M.L.H. in the same Seattle-based reference laboratory for all cohorts. Overall, 92 % of samples were run with the *orf73* assay, 5 % with the *orf26* assay and 3 % with the *orf73* and *T07.K12* multiplex assay.

### Statistical analysis

Sessions are defined as discrete periods of contiguous swab collection ≥14 days and separated in time from other observation periods. For participants contributing multiple sessions, the first and second sessions were included in the primary analysis. For each session, shedding rate was defined as number of swabs with HHV-8 DNA detection, divided by total swabs. Quantity of HHV-8 was log_10_ transformed for analysis and was assessed as maximum and median log_10_ copies/mL. We used Pearson correlations to evaluate the association of shedding rate with maximum and median log_10_ copy number within sessions.

To test the association of episode duration with time between episodes, we classified episodes as “short” or “long” by dichotomizing at the median length of all episodes and used log rank tests. First, we assumed episodes not observed to terminate were “long” and then then performed a sensitivity analysis reclassifying these episodes as “short”. We excluded Cameroonian samples from episode duration analysis since swabbing was not conducted daily.

Participants with two to five sessions were included in the longitudinal shedding analysis. The proportion of variability in shedding rate ascribed to the individual was evaluated with variance components.

## Footnotes

1 ORF26-F: 5’- AGC CGA AAG GAT TCC ACC AT-3’, ORF26-probe: ATG TGG TAC ACC AAC AGC TGC TGC, ORF26-R: 5’- TCC GTG TTG TCT ACG TCC AG-3’.

2 ORF73-F: 5'-CCA GGA AGT CCC ACA GTG TTC-3', ORF73-probe: FAM-CAT CCG GGC TGC CAG CAT TTG-TAMRA, ORF73-R: 5'-GCC ACC GGT AAA GTA GGA CTA GAC-3'.

3 T07.K12-F: 5'-TCC CCC ACC GAG TGC TT-3', T07.K12-probe: FAM-AAT GCG GAG AGG AAT G-MGB, T07.K12-R: 5'-GCA CGC GGT GTC AAC CA-3'.

## References

[1] Koelle DM, Huang ML, Chandran B, Vieira J, Piepkorn M, Corey L (1997). Frequent detection of Kaposi's sarcoma-associated herpesvirus (human herpesvirus 8) DNA in saliva of human immunodeficiency virus-infected men: clinical and immunologic correlates. J Infect Dis.

[2] Corey L, Brodie S, Huang ML, Koelle DM, Wald A (2002). HHV-8 infection: a model for reactivation and transmission. Rev Med Virol.

[3] Pica F, Volpi A (2007). Transmission of human herpesvirus 8: an update. Curr Opin Infect Dis.

[4] Johnston C, Orem J, Okuku F, Kalinaki M, Saracino M, Katongole-Mbidde E (2009). Impact of HIV infection and Kaposi sarcoma on human herpesvirus-8 mucosal replication and dissemination in Uganda. PLoS One.

[5] Shebl FM, Dollard SC, Pfeiffer RM, Biryahwaho B, Amin MM, Munuo SS (2011). Human herpesvirus 8 seropositivity among sexually active adults in Uganda. PLoS One.

[6] Simpson GR, Schulz TF, Whitby D, Cook PM, Boshoff C, Rainbow L (1996). Prevalence of Kaposi's sarcoma associated herpesvirus infection measured by antibodies to recombinant capsid protein and latent immunofluorescence antigen. Lancet.

[7] Pellett PE, Wright DJ, Engels EA, Ablashi DV, Dollard SC, Forghani B (2003). Multicenter comparison of serologic assays and estimation of human herpesvirus 8 seroprevalence among US blood donors. Transfusion.

[8] Goedert JJ, Kedes DH, Ganem D (1997). Antibodies to human herpesvirus 8 in women and infants born in Haiti and the USA. Lancet.

[9] Whitby D, Boshoff C, Hatzioannou T, Weiss RA, Schulz TF, Howard MR (1995). Detection of Kaposi sarcoma associated herpesvirus in peripheral blood of HIV-infected individuals and progression to Kaposi's sarcoma. Lancet.

[10] Soulier J, Grollet L, Oksenhendler E, Cacoub P, Cazals-Hatem D, Babinet P (1995). Kaposi's sarcoma-associated herpesvirus-like DNA sequences in multicentric Castleman's disease. Blood.

[11] Nador RG, Cesarman E, Chadburn A, Dawson DB, Ansari MQ, Sald J (1996). Primary effusion lymphoma: a distinct clinicopathologic entity associated with the Kaposi's sarcoma-associated herpes virus. Blood.

[12] Polizzotto MN, Uldrick TS, Hu D, Yarchoan R (2012). Clinical manifestations of Kaposi sarcoma herpesvirus lytic activation: multicentric Castleman disease (KSHV–MCD) and the KSHV inflammatory cytokine syndrome. Front Microbiol..

[13] Bray F, Ren JS, Masuyer E, Ferlay J (2013). Global estimates of cancer prevalence for 27 sites in the adult population in 2008. Int J Cancer.

[14] Casper C, Krantz E, Selke S, Kuntz SR, Wang J, Huang ML (2007). Frequent and asymptomatic oropharyngeal shedding of human herpesvirus 8 among immunocompetent men. J Infect Dis.

[15] Wald A, Zeh J, Selke S, Warren T, Ryncarz AJ, Ashley R (2000). Reactivation of genital herpes simplex virus type 2 infection in asymptomatic seropositive persons. N Engl J Med.

[16] Pauk J, Huang ML, Brodie SJ, Wald A, Koelle DM, Schacker T (2000). Mucosal shedding of human herpesvirus 8 in men. N Engl J Med.

[17] Taylor MM, Chohan B, Lavreys L, Hassan W, Huang ML, Corey L (2004). Shedding of human herpesvirus 8 in oral and genital secretions from HIV-1-seropositive and -seronegative Kenyan women. J Infect Dis.

[18] Schiffer JT, Wald A, Selke S, Corey L, Magaret A (2011). The kinetics of mucosal herpes simplex virus-2 infection in humans: evidence for rapid viral-host interactions. J Infect Dis.

[19] Schiffer JT, Abu-Raddad L, Mark KE, Zhu J, Selke S, Koelle DM (2010). Mucosal host immune response predicts the severity and duration of herpes simplex virus-2 genital tract shedding episodes. Proc Natl Acad Sci U S A.

[20] Perti T, Nyati M, Gray G, De Bruyn G, Selke S, Magaret A (2014). Frequent genital HSV-2 shedding among women during labor in Soweto. South Africa. Infect Dis Obstet Gynecol..

[21] Tanton C, Weiss HA, LeGoff J, Changalucha J, Clayton TC, Ross DA (2011). Patterns of herpes simplex virus shedding over 1 month and the impact of acyclovir and HIV in HSV-2-seropositive women in Tanzania. Sex Transm Infect.

[22] Mbopi-Kéou F-X, Grésenguet G, Mayaud P, Weiss HA, Gopal R, Matta M (2000). Interactions between herpes simplex virus type 2 and human immunodeficiency virus type 1 infection in African women: opportunities for intervention. J Infect Dis.

[23] Schoenfisch AL, Dollard SC, Amin M, Gardner LI, Klein RS, Mayer K (2011). Cytomegalovirus (CMV) shedding is highly correlated with markers of immunosuppression in CMV-seropositive women. J Med Microbiol.

[24] Lowhagen GB, Bergbrant IM, Bergstrom T, Voog E (1999). PCR detection of Epstein-Barr virus, herpes simplex virus and human papillomavirus from the anal mucosa in HIV-seropositive and HIV-seronegative homosexual men. Int J STD AIDS.

[25] Widmer IC, Erb P, Grob H, Itin P, Baumann M, Stalder A (2006). Human herpesvirus 8 oral shedding in HIV-infected men with and without Kaposi sarcoma. J Acquir Immune Defic Syndr.

[26] Nascimento MC, de Souza VA, Sumita LM, Freire W, Munoz F, Kim J (2007). Comparative study of Kaposi's sarcoma-associated herpesvirus serological assays using clinically and serologically defined reference standards and latent class analysis. J Clin Microbiol.

[27] Casper C, Krantz E, Taylor H, Dalessio J, Carrell D, Wald A (2002). Assessment of a combined testing strategy for detection of antibodies to human herpesvirus 8 (HHV-8) in persons with Kaposi's sarcoma, persons with asymptomatic HHV-8 infection, and persons at low risk for HHV-8 infection. J Clin Microbiol.

[28] Krone MR, Wald A, Tabet SR, Paradise M, Corey L, Celum CL (2000). Herpes Simplex Virus Type 2 Shedding in Human Immunodeficiency Virus- Negative Men Who Have Sex with Men: Frequency, Patterns, and Risk Factors. Clin Infect Dis.

[29] Schacker T, Hu HL, Koelle DM, Zeh J, Saltzman R, Boon R (1998). Famciclovir for the suppression of symptomatic and asymptomatic herpes simplex virus reactivation in HIV-infected persons. A double-blind, placebo-controlled trial. Ann Intern Med.

[30] Schacker TW, Conant M, Thoming C, Stanczak T, Wang Z, Smith M (2002). Imiquimod 5-percent cream does not alter the natural history of recurrent herpes genitalis: a phase II, randomized, double-blind, placebo-controlled study. Antimicrob Agents Chemother.

[31] Cattamanchi A, Saracino M, Selke S, Huang ML, Magaret A, Celum C (2011). Treatment with valacyclovir, famciclovir, or antiretrovirals reduces human herpesvirus-8 replication in HIV-1 seropositive men. J Med Virol.

[32] Cattamanchi A, Posavad CM, Wald A, Baine Y, Moses J, Higgins TJ (2008). Phase I study of a herpes simplex virus type 2 (HSV-2) DNA vaccine administered to healthy, HSV-2-seronegative adults by a needle-free injection system. Clin Vaccine Immunol.

[33] Zuckerman RA, Lucchetti A, Whittington WL, Sánchez J, Coombs RW, Zuñiga R (2007). Herpes simplex virus (HSV) suppression with valacyclovir reduces rectal and blood plasma HIV-1 levels in HIV-1/HSV-2-seropositive men: a randomized, double-blind, placebo-controlled crossover trial. J Infect Dis.

[34] Phipps W, Saracino M, Selke S, Huang ML, Jaoko W, Mandaliya K (2014). Oral HHV-8 replication among women in Mombasa. Kenya. J Med Virol..

[35] Mujugira A, Huang ML, Selke S, Drolette L, Magaret AS, Wald A (2015). High Rate of beta-Globin DNA Detection Validates Self-Sampling in Herpes Simplex Virus Shedding Studies. Sex Transm Dis.

[36] Gantt S, Cattamanchi A, Krantz E, Magaret A, Selke S, Kuntz SR (2014). Reduced human herpesvirus-8 oropharyngeal shedding associated with protease inhibitor-based antiretroviral therapy. J Clin Virol.

[37] Magaret AS, Johnston C, Wald A (2009). Use of the designation "shedder" in mucosal detection of herpes simplex virus DNA involving repeated sampling. Sex Transm Infect.

[38] Casper C, Redman M, Huang ML, Pauk J, Lampinen TM, Hawes SE (2004). HIV infection and human herpesvirus-8 oral shedding among men who have sex with men. J Acquir Immune Defic Syndr.

[39] Chandran B, Smith MS, Koelle DM, Corey L, Horvat R, Goldstein E (1998). Reactivities of human sera with human herpesvirus-8-infected BCBL-1 cells and identification of HHV-8-specific proteins and glycoproteins and the encoding cDNAs. Virology.

[40] Jerome KR, Huang ML, Wald A, Selke S, Corey L (2002). Quantitative stability of DNA after extended storage of clinical specimens as determined by real-time PCR. J Clin Microbiol.

[41] Zaniello B, Huang ML, Cheng A, Selke S, Wald A, Jerome KR (2015). Consistent viral DNA quantification after prolonged storage at ambient temperature. J Virol Methods..

[42] Shebl FM, Emmanuel B, Bunts L, Biryahwaho B, Kiruthu C, Huang ML (2013). Population-based assessment of kaposi sarcoma-associated herpesvirus DNA in plasma among Ugandans. J Med Virol.

[43] Magaret AS, Wald A, Huang ML, Selke S, Corey L (2007). Optimizing PCR positivity criterion for detection of herpes simplex virus DNA on skin and mucosa. J Clin Microbiol.

